# Urban Health Indicator Tools of the Physical Environment: a Systematic Review

**DOI:** 10.1007/s11524-018-0228-8

**Published:** 2018-04-16

**Authors:** Helen Pineo, Ketevan Glonti, Harry Rutter, Nici Zimmermann, Paul Wilkinson, Michael Davies

**Affiliations:** 1 Institute of Environmental Design and Engineering, Bartlett School of Environment, Energy and Resources, University College London, Central House, 14 Upper Woburn Place, London, WC1H 0NN UK; 2 Building Research Establishment, Bucknalls Lane, Garston, Hertfordshire, WD25 9XX UK; 3 School of Humanities and Social Sciences, University of Split, Split, Croatia; 4 Paris Descartes University, 12 Rue de l’École de Médecine, 75006 Paris, France; 5 Centre for Global Chronic Conditions, London School of Hygiene and Tropical Medicine, 15-17 Tavistock Place, London, WC1H 9SH UK; 6 Department of Social and Environmental Health Research, London School of Hygiene and Tropical Medicine, Keppel Street, London, WC1E 7HT UK

**Keywords:** Urban metrics, Built environment, Indicator, Indices, Policy, Urban health, Evidence, Urban planning, Healthy cities, Social determinants of health

## Abstract

Urban health indicator (UHI) tools provide evidence about the health impacts of the physical urban environment which can be used in built environment policy and decision-making. Where UHI tools provide data at the neighborhood (and lower) scale they can provide valuable information about health inequalities and environmental deprivation. This review performs a census of UHI tools and explores their nature and characteristics (including how they represent, simplify or address complex systems) to increase understanding of their potential use by municipal built environment policy and decision-makers. We searched seven bibliographic databases, four key journals and six practitioner websites and conducted Google searches between January 27, 2016 and February 24, 2016 for UHI tools. We extracted data from primary studies and online indicator systems. We included 198 documents which identified 145 UHI tools comprising 8006 indicators, from which we developed a taxonomy. Our taxonomy classifies the significant diversity of UHI tools with respect to topic, spatial scale, format, scope and purpose. The proportions of UHI tools which measure data at the neighborhood and lower scale, and present data via interactive maps, have both increased over time. This is particularly relevant to built environment policy and decision-makers, reflects growing analytical capability and offers the potential for improved understanding of the complexity of influences on urban health (an aspect noted as a particular challenge by some indicator producers). The relation between urban health indicators and health impacts attributable to modifiable environmental characteristics is often indirect. Furthermore, the use of UHI tools in policy and decision-making appears to be limited, thus raising questions about the continued development of such tools by multiple organisations duplicating scarce resources. Further research is needed to understand the requirements of built environment policy and decision-makers, public health professionals and local communities regarding the form and presentation of indicators which support their varied objectives.

**Electronic supplementary material:**

The online version of this article (10.1007/s11524-018-0228-8) contains supplementary material, which is available to authorized users.

## Introduction

Both the global increase in non-communicable diseases and improved understanding of the social determinants of health have contributed to an increased awareness of the influence of built environment policies on health and wellbeing [1–3]. Estimates vary, but recent research attributes 23% of global deaths to the environments in which people live [4]. The urban environment, including air pollution, noise, housing and transport, plays a significant role in people’s health, and improvements should involve collaboration between health and built environment professionals [2, 5]. Other social determinants, such as employment and education, are also influenced by urban planners, increasing the importance of their work for population health [6]. Municipal built environment practitioners can improve health through policies and decisions which identify the need for and design of new infrastructure, development and regeneration programmes.

Urban health indicator (UHI) tools seek to provide built environment policy and decision-makers with information to develop policies, make decisions and monitor impacts. These metrics can demonstrate the impact of the built environment on health and expose health inequalities within cities. Urban health is a complex system with many interconnected parts [7–10] which UHI tools attempt to simplify for policy-makers [11]. The range of potential uses of indicators by municipal government is vast. Further to the above-mentioned uses, indicators are also employed to [12–18]:

- Benchmark progress at local, regional, national or international levels
- Set targets for improvement
- Demonstrate performance to residents
- Prioritise funding allocation/bid for funding
- Act as an ‘early warning’ of potential problems
- Involve the public in prioritisation and definition of policy goals
- Identify strengths and weaknesses in a community

The intended use of indicator tools is likely to inform their composition and characteristics, elements which are often represented in a taxonomy [19]. Taxonomies have been developed for mental health and ecological indicators by identifying and classifying user requirements such as spatial scale and decision-making context [20, 21]. Yet, research addressing how indicators are used and how they can be standardised is missing, providing two main reasons why an improved understanding of UHI tool characteristics and an associated taxonomy may help indicator producers and users.

First, indicator researchers have tended to focus on the development and validation of indicator tools, rather than investigating how such tools are used by policy- and decision-makers [15]. The production of new indicator tools is often a duplication of previous research efforts. However, there is recognition that locally developed tools may increase acceptability and allow for tailoring of indicators to local needs [19, 22, 23]. In fact, some have argued that the process of indicator development is at least as important in achieving change as the eventual use of indicators [16, 22]. Increased understanding of the characteristics of UHI tools which meet the needs of policy and decision-makers could reduce wasted efforts by indicator producers and increase usability for indicator users.

Second, despite the large amount of research on indicator development, there is still a lack of consensus on how to measure the urban environment’s impact on health and related concepts. Standardising the development of urban health indicators is a topic of ongoing debate [23, 24]. Despite the large number of UHI tools already available, researchers continue to contribute new international indicator sets whilst implicitly supporting greater standardisation (see [25, 26]). Salvador-Carulla and colleagues argued that there is a lack of international consensus on indicators and that indicator tools ‘lack adequate semantic interoperability’ [20]. A taxonomy which describes the general characteristics of UHI tools would provide a useful step toward standardisation, resulting in reduction of duplicated efforts and easier identification of appropriate UHI tools.

To our knowledge, there exist three reviews of relevant indicators. The Prasad et al. systematic review of urban health metrics highlighted the lack of available data for metrics in low and middle income countries and questioned the translation of evidence gained through using such metrics into policy and decision-making [27]. Rothenberg et al. conducted a non-systematic review of urban health indicators and metrics which found that indicator sets focus on large-area comparisons (nations, states) and that small-area comparisons (cities, neighborhoods) are relatively underdeveloped [19]. They also observed similarity in the domains measured across compilations. The Badland et al. review of urban liveability indicators for the Australian urban planning policy context found inconsistency in how domains were measured, a relative lack of validated indicators and a lack of information on how to apply indicators to inform urban policy and practice [9].

This systematic review examines a specific type of indicator compilation which could inform municipal built environment policy and decision-makers about the social determinants of health, defined as ‘urban health indicator tools’. The review has two distinct parts, as outlined in a previously published protocol [28, 29]. Part A seeks to conduct a census of UHI tools to describe their characteristics and develop a taxonomy of such tools. Part B seeks to explore the perceptions and use of UHI tools by built environment policy and decision-makers. Both parts examine how UHI tools address the complexity of urban health and how this complexity affects policy and decision-making. This paper reports the findings of Part A.

## Methods

The protocol for this review was published in *Systematic Reviews* including a completed PRISMA-P checklist [28]. From January 27, 2016 to February 24, 2016, we searched seven bibliographic databases using search terms and MeSH subject headings related to (1) the urban environment, (2) health and related concepts and (3) indicators. We conducted Google Advanced searches on six practitioner websites and the internet using specified search terms in line with the search strategy for databases. There was no date restriction on database searches. We hand-searched four key journals with date restrictions of 3 to 5 years depending on the relevance of articles found and the number of volumes per year. Table 1 shows the sources searched for the review.

**Table 1:** Databases, websites and journals searched for the review, including years hand-searched for journals

Source type	Source
Bibliographic databases	Applied Social Sciences Index and Abstracts (ASSIA)
	Campbell Library
	Embase
	Medline
	Scopus
	Social Policy and Practice
	Web of Science Core Collection (includes the Social Sciences Citation Index)
Websites	Town and Country Planning Association (UK)
	Royal Town Planning Institute (UK)
	Planning Institute of Australia
	American Planning Association
	Built Environment and Public Health Clearinghouse (USA)
	World Health Organization Europe, Urban Health, Healthy Cities
Hand-searched journals	Annual Review of Public Health (5 years)
	Social Science and Medicine (3 years)
	BMC Public Health (1 year)
	Social Indicators Research (3 years)

## Eligibility Criteria

A UHI tool was defined as ‘a collection of summary measures about the physical urban environment’s contribution to human health and wellbeing’ [28]. A combination of indicators can be referred to as a set, compilation, collection or tool [19, 30, 31]. We selected the term ‘tool’ because it reflects a utility or intention to support policy and decision-making. Tools which sought to measure the related concepts of quality of life (QOL), wellbeing and liveability were also included. During the screening stage, we decided to include tools which measured the impact of the physical urban environment on walkability/physical activity (PA) as this is an important contribution of the built environment toward promoting good health [32, 33]. Any UHI tool which met the definition was referred to in peer-reviewed or grey literature documents (including websites) and was published in English was included in the review. UHI tools needed to measure at least two different aspects of the physical urban environment to be included (e.g. housing and air quality).

All documents were screened by the principal investigator (HP) and a random sample of 10% of documents were screened by a second reviewer (KG) at the title and abstract and full paper screening stages. Differences were resolved through discussion. Eppi-Reviewer software was used to manage all documents and screening.

## Data Extraction and Analysis

The name of each UHI tool was entered as a search term in Google to find additional information and sources. Data were extracted from the original source wherever possible. Characteristics of UHI tools were extracted and analysed in Excel. The characteristics extracted were informed by a scoping review (reported in the protocol) and included four additional points that were not listed in the protocol:

- Topic: concept that the UHI tool measured (e.g. health or liveability)
- Main source of data (e.g. municipal datasets or resident surveys)
- Indicator type: subjective or objective (as defined in Lowe et al. [36 p. 136])
- Whether the tool had been used beyond research

The last point was informed by the Google search of each indicator tool. If this search produced evidence of case studies, policy documents or other uses beyond the original research paper, this was marked as ‘used beyond research’. The others were marked as ‘unknown’.

We modified approaches used by Salvador-Carulla et al. [20] and Wardrop et al. [21] to develop our taxonomy. Salvador-Carulla and colleagues developed key topics for their taxonomy by reviewing published literature and indicator lists. Then they discussed these topics with expert groups. Wardrop and colleagues developed their taxonomy on the basis of characteristics of environmental indicators which would be useful for environmental managers using a survey of government officials. We combined and modified these approaches. We used relevant literature [9, 19, 27] and the data gathered in the review to identify five key characteristics of UHI tools for built environment professionals: spatial scale, purpose, topic, scope and format. These became the highest level category within the taxonomy, denoted as ‘class’. Data were extracted on each of the five classes. The second order in the taxonomy, ‘sub-class’, was developed during the analysis of data extracted in the review, noting differences within each class and categorising these in an iterative process. UHI tools may have characteristics from multiple sub-classes (they are not mutually exclusive). Indicator domains (listed as sub-classes under ‘scope’) were selected using a set of domains identified from previous reviews [9, 19]. For analysis purposes, all 8006 indicators were standardised to this list of domains. It is possible to divide these domains into smaller groups (e.g. chronic diseases and injuries could be sub-domains under the domain of health outcomes).

During data analysis the term neighborhood was grouped with other sub-city spatial scales including ward and district. Lower than neighborhood scales were also grouped together, representing street or household scale for example. Given variation in the meaning of terms like ‘district’ or ‘post-code’, scales were assigned on the basis of authors’ descriptions.

UHI tools report data, and are available for use, at different spatial scales. These were reported using three terms: spatial scale, general geography and specific geography. Spatial scale referred to the level of data aggregation for which the tool reported indicator data. General geography referred to the geographical scales in which a particular UHI tool could be accessed (such as a city, county or state). Specific geography added a place name to that general term. For example, the U.S. Centers for Disease Control and Prevention’s ‘Environmental Public Health Tracking Network’ covered the whole country and allowed users to select indicator data at the county and zip code scales (with comparison of state averages as well) [34]. The data for this UHI tool was thus extracted as:

- Spatial scale: multiple (county, zip code)
- General geography: country
- Specific geography: USA

## Results

**Fig. 1:**
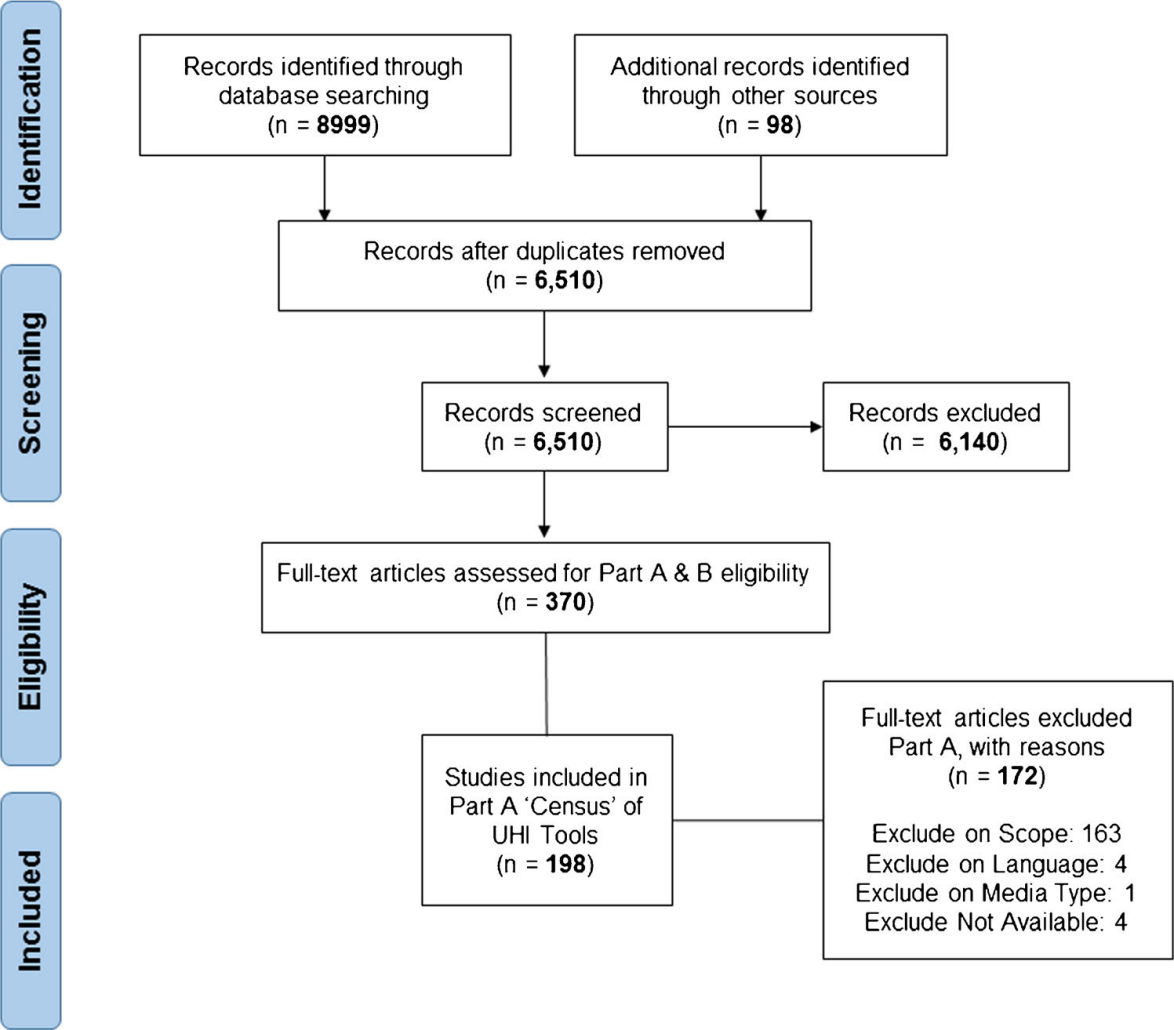
Flow of documents through the review, following PRISMA reporting style [35]

The flow of documents through the review is shown in the PRISMA diagram (Fig 1). There were 9097 records identified in the database, internet and journal searches. After duplicates were removed, 6510 titles and abstracts were screened. Of these, 370 were included in a full-text review. Finally, 198 documents were included in the Part A census of UHI Tools. These documents referred to 145 separate urban health indicator tools (Appendix 1) which comprised 8006 indicators.

## Taxonomy of UHI Tools

**Fig. 2:**
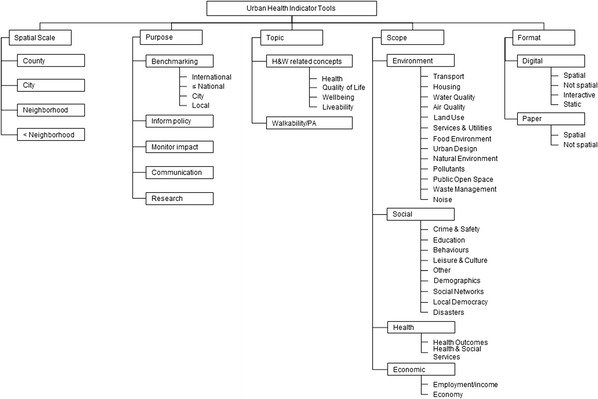
Taxonomy of urban health indicator tools. H&W, health and wellbeing; PA, physical activity

Figure 2 shows our taxonomy with five classes: spatial scale, purpose, topic, scope and format. In this section, we present the taxonomy and review each class and its sub-classes.

### Spatial Scale

**Fig. 3:**
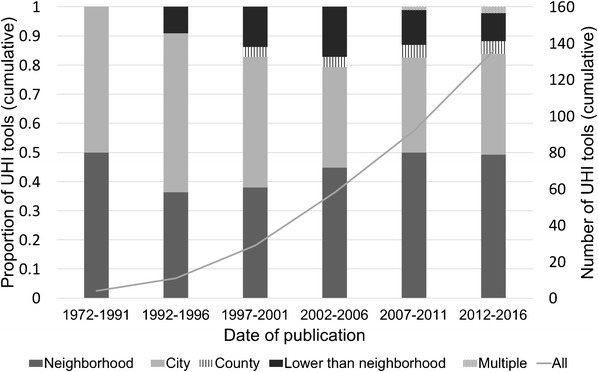
Change over time of proportion of UHI tools by spatial scale compared with cumulative growth of UHI tools. N.B. Missing data for 9/145 UHI tools: 7 did not report a date of publication and 2 did not report spatial scale

**Fig. 4:**
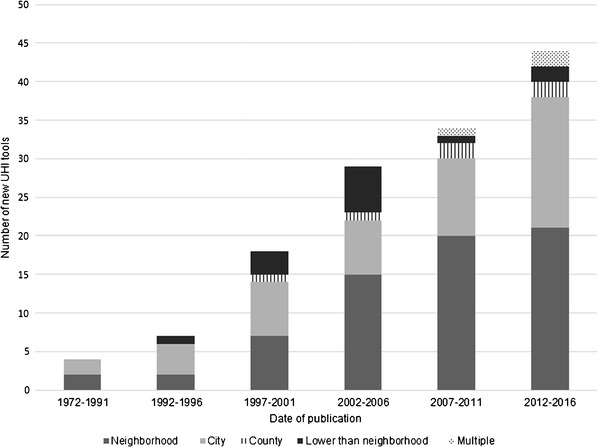
Number of new UHI tools by spatial scale. N.B. Missing data for 9/145 UHI tools: 7 did not report a date of publication and 2 did not report spatial scale

Of the UHI tools included in this review, 59.3% (86/145) measured data at the neighborhood scale or lower. Over time, the proportion and number of UHI tools which present data at the neighborhood scale and lower has increased (Figs. 3 and 4).

### Purpose

**Fig. 5:**
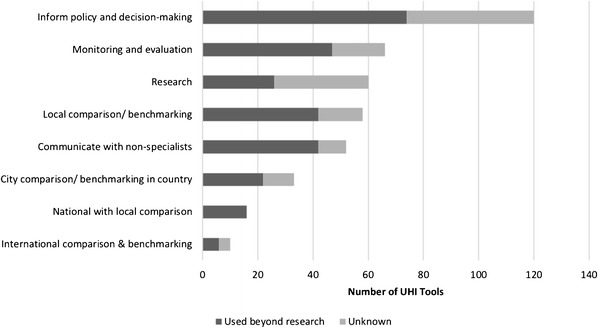
Number of UHI tools in each stated purpose categorised by those which were used beyond research and ‘unknown’

Of UHI tools, 82.8% (120/145) stated that part of their purpose was to inform policy and decision-making (Fig. 5). Monitoring and evaluation (45.5%, 66/145), research (41.4%, 60/145), local comparison/benchmarking (40.0%, 58/145) and communicating with non-specialists (35.9%, 52/145) were also commonly stated goals of UHI tools. The majority of tools (54.5%, 79/145) were found to be used beyond research.

### Topic

The concepts of QOL, wellbeing and liveability are closely related to human health and their definitions overlap significantly. Table 2 lists a selection of definitions or explanations of these concepts which were identified in the systematic review (or citations found therein) and demonstrates overlaps between the ways in which these concepts were defined.

**Table 2:** Definitions and explanations of quality of life, liveability and wellbeing concepts from selected papers included in the systematic review or citations found therein

Concept	Definition
Quality of life	‘The wellbeing of individuals within the context of their environment’ [36]
	‘An individual’s happiness or satisfaction with life and environment including needs and desires and other tangible and intangible factors which determine overall wellbeing’ [37, 38]
Liveability	‘Closely aligned with the social determinants of health’ [9]
	‘The human requirement for social amenity, health and wellbeing and it includes both individual and community wellbeing’ [39]
Wellbeing	“Associated with concepts such as happiness, life satisfaction and social capital, all of which fall under the rubric of a ‘social quality of life’” [40]
Community wellbeing	‘Reflect a community’s health status and its basic quality of life’ [40]

**Fig. 6:**
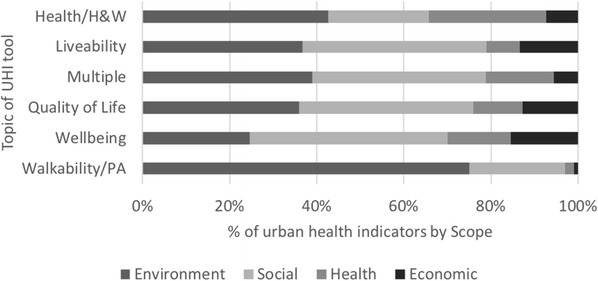
Scope of indicators across UHI tool topics. PA, physical activity; H&W, health and wellbeing

Analysis of the indicator domains showed that there is some homogeneity of scope across tools which measure different health-related concepts, with the exception of walkability/PA tools (Fig. 6). Each topic area (excluding walkability/PA) measured a similar proportion of environmental (18.2– 44.1%), social (23.2– 41.8%), health (7.6– 27.7%) and economic indicators (7.9– 13.5%). Given the significant difference of scope in the walkability/PA tools (75.1% environmental indicators), this topic area was noted as a separate sub-class in the taxonomy to the more similar health-related concepts.

**Fig. 7:**
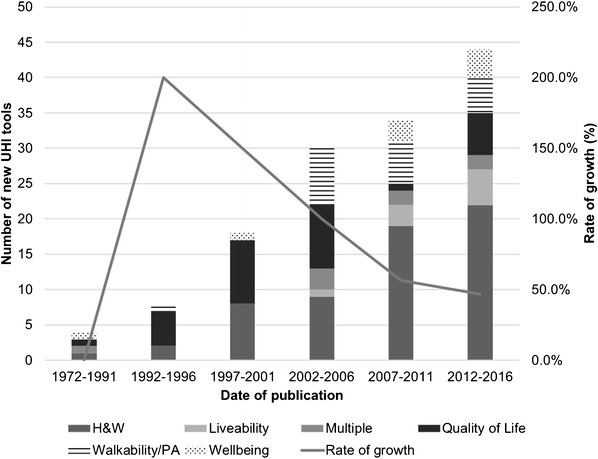
Date of publication of UHI tools by topic area and rate of growth. N.B. Missing data for 7/145 UHI tools which did not report a date of publication

Health and wellbeing (H&W) (45.5%, 66/145) and QOL (22.1%, 32/145) were the most common topic areas across the tools. Walkability/PA tools (13.8%, 20/145) are a relatively recent addition in urban health metrics (Fig. 7). Bradshaw’s Walkability Index from 1993 was the first example, with the remainder produced from 2002 [41]. There were only four UHI tools found between 1972 and 1991, with the number of new tools increasing 14 times by the end of 2006. The rate of growth was between 100 and 200% between 1972 and 2006 (Fig. 7). In the last decade, the growth rate has slowed to between 46.8 and 56.7%.

Table 3 shows a breakdown of domains across topic areas. Between four to seven of the top ten domains for health and wellbeing appear in the top ten for the other topic areas, illustrating the overlap of domains across each topic. The least similar topic is walkability/PA which only shares four domains with the H&W topic.

**Table 3:**
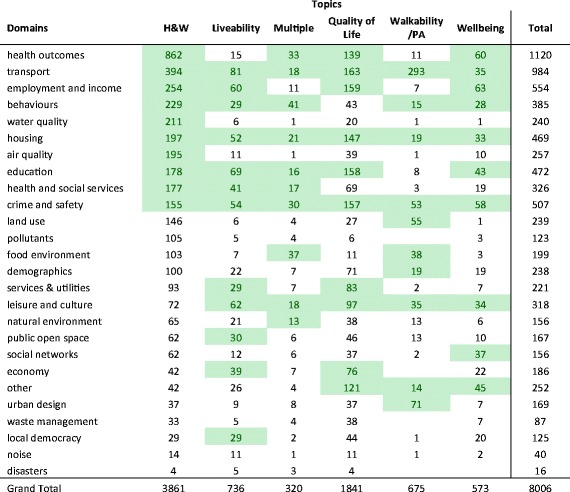
Number of indicators in each domain across UHI tool topic areas, sorted by H&W

Top 10 domains are highlighted in green for each UHI topic area

*H&W* health and wellbeing, *PA* physical activity

### Scope

Indicators under the scope of environment made up the largest portion (41.9%, 3351/8006). Table 4 shows the four scopes with each of their composite domains and the number of indicators in each.

**Table 4:** Indicator domains grouped by scope across all UHI tools (total of 8006 indicators)

**Category**	**Domains**	**Number of indicators**
**Environment**		
	Transport	984
	Housing	469
	Air quality	257
	Water quality	240
	Land use	239
	Services and utilities	221
	Food environment	199
	Urban design	169
	Public open space	167
	Natural environment	156
	Pollutants	123
	Waste management	87
	Noise	40
	**Category total**	**3351**
**Social**		
	Crime and safety	507
	Education	472
	Behaviours	385
	Leisure and culture	318
	Other	252
	Demographics	238
	Social networks	156
	Local democracy	125
	Disasters	16
	**Category total**	**2469**
**Health**		
	Health outcomes	1120
	Health and social services	326
	**Category total**	**1446**
**Economic**		
	Employment and income	554
	Economy	186
	**Category total**	**740**

UHI tools measured between 3 and 286 individual indicators (average 56). Across the 145 UHI tools, 3 did not report the full list of indicators.

### Format

**Fig. 8:**
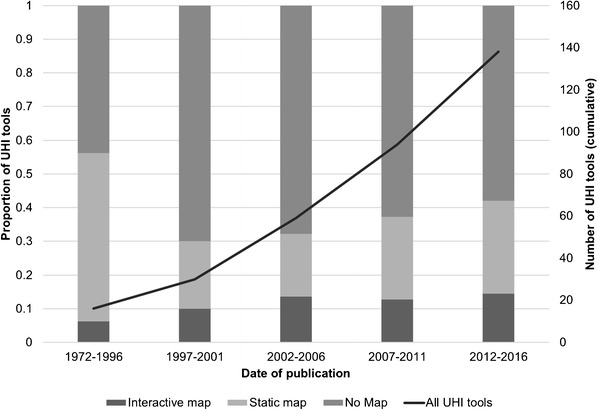
Proportion of UHI tools which display data on static and interactive maps over time, compared with the cumulative growth of all UHI tools. N.B. Missing data for 7/145 UHI tools which did not report a date of publication

Of UHI tools, 44.1% (64/145) displayed data on static or interactive maps, and from 1997, the number and proportion of these tools has grown (Fig. 8). Interactive maps allowed users to select indicators and/or locations to be mapped through an online dashboard. Nearly all (96.0%, 24/25) of the UHI tools which had an interactive mapping function intended to inform policy and decision-making. Examples include ‘Peg Wellbeing Indicators’ and the health profiles on the ‘Plan for a Healthy Los Angeles’ website [42, 43]. Three-quarters of these interactive UHI tools (76.0%, 19/25) displayed data at the neighborhood scale. Most of these tools (92.0%, 23/25) also allowed local comparison and benchmarking across other neighborhoods and counties.

## Other Characteristics of UHI Tools

This portion of the results section presents additional characteristics of UHI tools which were not used to form the taxonomy. See the protocol for the full list of items extracted and the Supplementary Material section for additional details and results.

**Fig. 9:**
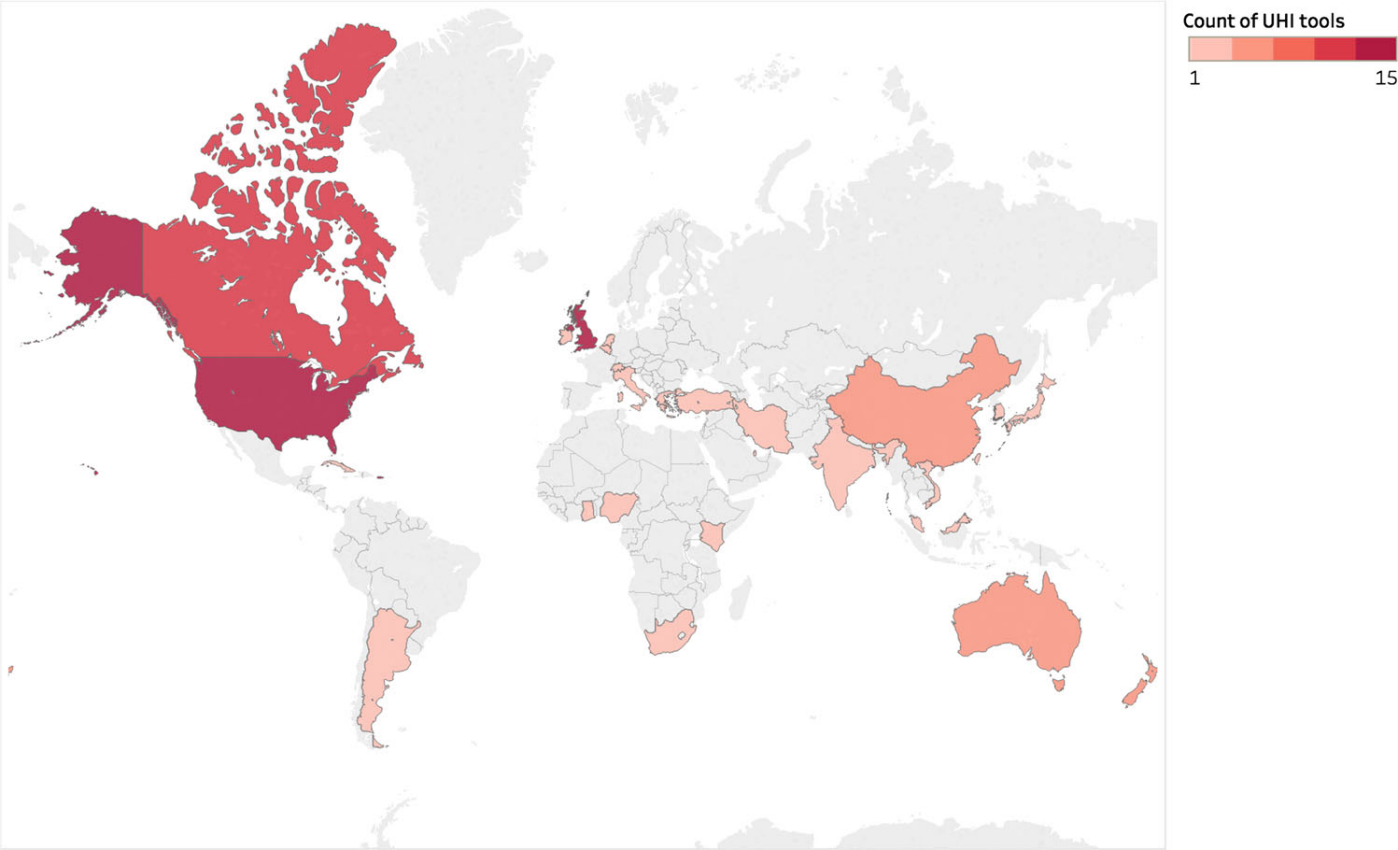
Location of UHI tools internationally. N.B. Tools which apply in more than one country are not shaded

Of the tools, 37.9% (55/145) were available at the city-scale with national systems following closely behind (31.0%, 45/145). Many tools were available internationally (19.3%, 28/145). Tools were found for 28 individual countries (Fig. 9). In addition, there were 28 international tools (i.e. could be used in any country) and 4 European tools.

Research institutions were the largest producer of UHI tools (54.5%, 79/145). Many of the tools produced by research institutions were not found to have been used beyond research (62.7%, 37/59). The funding source was often not stated (46%, 67/145). Where reported, the largest funder of UHI tools was government (17.9%, 26/145). Of the UHI tools, 86.9% (126/145) reported some information about the methodology. Evidence which informed the methodology or indicator selection was reported in 99/145 cases (68.3%). Peer-reviewed literature was the largest primary source of evidence used in 52.4% (76/145) of tools. The majority of tools (57.9%, 84/145) used existing datasets from multiple organisations to measure the indicators.

A significant number of tools referred to complexity in the methodology (43%, 63/145). The word complexity was mentioned in 128 instances covering multiple topics, including:

- Indicators/indices can simplify or mask the complexity of the concepts being measured
- The urban environment impact on health and behaviour is complex
- Measuring the urban environment’s impact on health is complex
- The process of policy and decision-making is complex

Eleven UHI tools stated that indicators or composite indices can simplify the complexity of the concepts being measured. In relation to the City of Winnipeg Quality of Life Indicators, Hardi and Pintér explained: ‘[i]ndicators are used to simplify information about complex phenomena, such as sustainable development or, in this case, QOL, in order to make communication easier and quantification possible’ [11]. This was contrasted by the opposing view that indicators/indices can mask complexity (two instances). The authors of the London Quality of Life Indicators stated: ‘[a]lthough the Commission have sought to identify and report on 20 headline indicators, to constitute a popular ‘barometer’ for London’s quality of life, it is clear that single figure measures can mask a much more complex situation’ [44].

Three UHI tools referred to the complex process of policy and decision-making, sometimes in recognition that indicators may not inform policy due to this complexity. For example, Hunt and Lewin commented that ‘policy action may not easily follow the identification of environmental health problems [through indicators], which is due both to the large numbers of other factors that also affect health and to the complexity of the policy process’ [45].

UHI tools rarely explained strategies used to help account for complexity. Feneri et al. used Multi-criteria Decision Analysis to ‘conceptualize the complex issue of evaluating quality of life’ [46]. They specified the use of Analytical Hierarchy Process to prioritise indicators. The AARP Livability Index used a high number of indicators to address complexity, stating: ‘[s]imple questions about livability [sic] can have complex answers. This is why the index includes a large number of metrics’ [47].

## Discussion

This review identified great diversity in the purpose and characteristics of urban health indicator tools making it difficult to draw simple conclusions. However, the review generated novel findings about UHI tools as they relate to the needs of built environment policy and decision-makers. Our taxonomy demonstrates the importance of considering users’ needs when developing indicator tools to ensure they can be used to support built environment practitioners. Our main findings are summarised here with implications discussed below. First, we found that the proportion of tools with data aggregation/measurement at the neighborhood and lower scale and presentation of data via digital interactive maps have both increased over time. Second, we highlighted that the majority of UHI tools *intend* to inform policy and decision-making, yet it is unclear whether a significant number achieve this aim. Third, we found that the majority of UHI tools are evidence-based and therefore provide a potential route from research through to policy. Fourth, we have explored the nature of how UHI tool methodologies address complexity, identifying specific strategies. Finally, we have shown that there is a degree of similarity in the domains measured across UHI tool topics.

In comparison to existing reviews of indicators which measure the urban environment’s impact on health, this review casts a wider net by including measures of health, QOL, liveability, wellbeing, and walkability/physical activity. This has enabled a detailed analysis of a large number of indicator tools and their respective characteristics, including 8006 individual indicators. The review was limited to English language publications, potentially excluding many UHI tools from non-English language countries. The method used to classify whether a tool had been used beyond research was simplistic and may have underestimated those tools which were indeed used beyond research.

The increasing number of UHI tools with data aggregation at neighborhood or lower scale is of significance for built environment policy and decision-makers. In 2002, Talen questioned the usefulness of indicators to inform urban planning because the majority were comparing cities (inter-city) rather than neighborhoods (intra-city) [48]. Neighborhood and lower scale of measurement or data aggregation is more appropriate for identifying health inequalities and environmental deprivation which may contribute to poor health [49]. Indicators at this scale can be used to inform neighborhood development/regeneration policies and monitor the impact of these over time. Data visualisation is also frequently noted as a helpful feature of UHI tools for built environment policy and decision-makers, particularly in relation to displaying data on maps [19, 27]. The growing numbers of UHI tools which present data on interactive maps at the neighborhood or lower scale are likely to be a powerful source of information for built environment policy and decision-makers.

A number of tools (31.7%, 46/145) did not explain the evidence used for indicator selection, creating questions over the suitability of their use in policy and decision-making. Although the validity of individual indicators (association between exposure and outcome) was not assessed by this review, the range of methods for selecting indicators demonstrated that this process was not always informed by evidence about environmental exposures and health effects. Badland et al. called for further research about the validity of indicators within UHI tools (specifically in relation to liveability indicators) [9]. However, we would suggest that there is a large selection of validated indicators in the published literature and research efforts may be better directed toward understanding how existing indicators are used to guide the policy and decision-making process.

The distinction of whether UHI tools are used beyond research is of interest when considering transfer of research knowledge to practitioners. We were unable to confirm whether 45/120 tools (37.5%) which intended to inform policy/decision-making achieved this aim. There could be a delay between research and use or this may also point to other knowledge translation issues. UHI tool producers should consider the needs of their audience and may benefit from wider strategies to increase research use by policy and decision-makers (see [50]). The apparent low use of many UHI tools leads us to consider whether greater standardisation of indicators is required rather than development of new indicator tools.

Standardisation of UHI tools may be aided by our finding that there is significant overlap across domains measuring health-related topics such as QOL, liveability and wellbeing. Rothenberg et al. also found similarities in indicator domains across urban health indicator compilations [19]. Guidance on developing indicators of health and the determinants of health is supported by specific frameworks (e.g. DPSEEA) that emphasise the requirement for an evidence-based, often causal relationship between environmental exposures and specific health outcomes [24, 51]. This formality may increase the acceptability of a standardised set of indicators. However, lack of consensus over how to define and measure related topics like QOL, wellbeing and liveability (despite similarity in existing UHI tools) may mean that standardisation for these topics is harder to achieve.

A standardised set of global indicators would mean that rather than developing new UHI tools, researchers and practitioners could choose from an internationally published set of evidence-based indicators. Local selection of indicators would likely be based on data availability, health priorities and community opinion. The WHO’s Urban Health Index provides methods for local public health data analysts to produce local indices (including instructions for mapping the results) [52, 53]. Such a tool is valuable to avoid duplicated effort when selecting appropriate indicator aggregation methods. However, we suggest that a set of global evidence-based indicators, which the WHO’s Urban Health Index currently lacks, would be of great value to local indicator projects. Given that many global UHI tools are already available, a standardised set would need to be widely promoted and supported to achieve impact and avoid further duplication of effort. Further research is needed to determine whether a standardised set of urban health indicators could be promoted globally and accepted locally (such as the Sustainable Development Goals).

Whilst some indicator producers recognised that indicators could help explain complex phenomena, other authors noted that they may not be effective at influencing a complex policy and decision-making process. This topic will be explored further in a subsequent paper related to this systematic review which will synthesise qualitative data from studies exploring the use of UHI tools in the built environment policy and decision-making process.

Observing the similarity across indicator measures, there is a question about whether some data are included simply because they are easy to measure (or commonly measured as a part of routine statistics), whilst other more difficult topics are excluded. For example, although noise is known to impact multiple health outcomes [54], it is less frequently measured in UHI tools, reflecting the difficulty of measuring this exposure. This is an area for further investigation. The growth of city datasets emerging from open data initiatives may increase the need for indicators to help interpret and make sense of data. This may also support increased small-scale spatial comparisons, improving usability by built environment policy and decision-makers. New data from smartphones, social media and other sources are also likely to increase available datasets for UHI tools and may be a useful way to increase citizen participation in generating and evaluating indicator data.

## Electronic supplementary material

ESM 1 (DOCX 122 kb)

## Appendix 1

**Table 5:** All UHI tools identified in the review with their characteristics relating to the five classes in the taxonomy

Tool/index	Topic	Spatial Scale (of data aggregation)	Place (of tool availability)	Format		Scope (no. of indicators)				Purpose							
				Interactive map	Static map	Economic	Environment	Health	Social	International benchmarking	City (or in country) benchmarking	National with local benchmarking	Local benchmarking	Inform policy and decision-making	Communicate with Non-specialists	Monitoring /evaluation	Research
2011 Livable City Index [55]	H&W	City	China			8	73	5	33		Y			Y			
Abbreviated Neighborhood Environment Walkability Scale (ANEWS) [56]	Walkability/PA	Neighborhood	International			0	33	0	21								Y
Active Neighborhood Checklist [57]	Walkability/PA	<Neighborhood	USA			0	43	0	7					Y	Y		Y
Active Transportation and Health Indicators [58]	H&W	City and neighborhood	Peterborough, Canada		Y	3	79	12	9				Y	Y	Y	Y	
Activity-Friendly Index [59]	Walkability/PA	City and neighborhood	Toronto, Canada		Y	0	4	0	1				Y	Y			
American Fitness Index [60]	Walkability/PA	City	USA			3	9	9	22		Y	Y		Y	Y	Y	
ANQoLHP Neighborhood Health Index [61]	H&W	City and neighborhood	Atlanta, GA, USA	Y		0	2	6	0				Y	Y	Y	Y	
ANQoLHP Neighborhood Quality of Life Index [36]	Quality of life	City and neighborhood	Atlanta, GA, USA	Y	Y	1	7	1	2				Y	Y	Y	Y	
Baltimore Neighborhood Indicators Alliance, Vital Signs [62]	Quality of life	City and neighborhood	Baltimore, MD, USA	Y		27	46	15	74				Y	Y	Y	Y	Y
Border Observatory Project [63]	Quality of life	City	USA and Mexico			5	16	5	18	Y	Y			Y		Y	
Bristol Quality of Life Indicators [64]	Quality of life	City and neighborhood	Bristol, UK	Y	Y	4	42	12	89				Y	Y		Y	
British Colombia Atlas of Wellness [65]	Multiple	Multiple	British Columbia, Canada		Y	7	22	33	64		Y		Y	Y	Y		
Buffalo City QOL Survey [66]	Quality of life	City and neighborhood	Buffalo City, South Africa			6	27	3	21				Y	Y		Y	
Built Environment Assessment Tool [67]	H&W	<Neighborhood	International			0	71	0	6					Y	Y	Y	
Built Environment Site Survey Checklist, BESSC [68]	H&W	<Neighborhood	England			0	18	0	9								Y
CANVAS (Computer-Assisted Neighborhood Visual Assessment System) [69]	H&W	<Neighborhood	International			0	134	1	27								Y
Caya Hueso Urban Ecosystem Health Indicators [70]	H&W	Neighborhood	Habana, Cuba			5	17	11	16					Y	Y	Y	
Child Opportunity Index [71]	H&W	Neighborhood	USA	Y	Y	5	6	1	7		Y	Y	Y	Y	Y	Y	Y
Childhood wellbeing indicators [72]	Wellbeing	Neighborhood	International			3	5	5	18				Y				
Children’s Environmental Health Indicators [73]	H&W	Not specified	International			5	18	25	6					Y	Y	Y	Y
Christchurch City Health and Wellbeing Profile [74]	H&W	City	Christchurch, New Zealand		Y	4	13	13	18					Y	Y		
City Ecosystem Health Index [75]	H&W	City	Chongqing, China			4	11	2	2					Y			
City Livability Index [76]	Liveability	City	China			5	4	3	2		Y			Y			
City of Melbourne Urban Health Profile metrics [77]	H&W	City and neighborhood	Melbourne, Australia			1	4	11	16				Y	Y		Y	
City of Winnipeg Quality-of-Life Indicators [11]	Quality of life	City	Winnipeg, Canada			14	25	6	15					Y		Y	
Coalitions Linking Action and Science for Prevention (CLASP) Tool [78]	Walkability/PA	Neighborhood	Canada	Y	Y	4	31	2	19				Y	Y			
Colorado Health Indicators [79]	H&W	County	Colorado, USA	Y	Y	13	23	91	60			Y	Y	Y	Y		Y
Combined Environmental Stressor’s Exposure (CENSE) Tool [80]	H&W	Neighborhood	International			0	7	0	0					Y			
Communities Count [81]	H&W	County	King County, WA, USA		Y	49	25	66	113			Y	Y	Y	Y	Y	
Community Health and Equity Index [82]	H&W	Neighborhood	Los Angeles, CA, USA		Y	3	16	7	3				Y	Y		Y	
Community Health Environment Scan Survey (CHESS) [83]	H&W	Neighborhood	International			0	28	0	24					Y			Y
Community Health Status Indicators [84]	H&W	County	USA	Y		2	6	28	7		Y	Y	Y	Y	Y		
Community Healthy Living Index [85]	H&W	Neighborhood	USA			0	23	1	12					Y	Y		
Community Indicators Victoria [86]	H&W	≥ City	Victoria, Australia	Y		13	80	14	83				Y	Y	Y	Y	Y
Community Well-Being Index (A) [87]	Wellbeing	City	Korea			14	11	12	47		Y			Y			
Community Well-Being Index (B) [88]	Wellbeing	City	Flint, MI, USA			11	25	4	64					Y		Y	
Community Wellbeing Questionnaire [13]	Wellbeing	Neighborhood	International			3	11	2	29					Y		Y	Y
Core Environmental Health Indicators in Lucknow and Calcutta [45]	H&W	Neighborhood	India			0	9	0	1					Y	Y		
County Health Rankings [89]	H&W	County	USA	Y		3	7	19	8				Y	Y	Y	Y	
DECAMB Programme Indicators for the Urban Environment [90]	Quality of life	Not specified	Italy			0	6	1	4					Y			Y
Edmonton LIFE: Local Indicators For Excellence [91]	Quality of life	City	Edmonton, Canada			12	11	10	21		Y			Y	Y	Y	
Environmental Index [92]	H&W	City	Netherlands		Y	0	4	0	0					Y			
Environment Health Sustainability (EHS) Index [93]	H&W	City	USA			3	55	9	4					Y	Y	Y	
Environmental Health Basic Exposure Survey [94]	H&W	Neighborhood	Baltimore, MD, USA		Y	0	12	9	1					Y			Y
Environmental Health Indicators New Zealand (EHINZ) [95]	H&W	City and neighborhood	New Zealand	Y		1	32	16	7		Y	Y		Y		Y	
Environmental Profile of a Community’s Health (EPOCH 1) [96]	Walkability/PA	Neighborhood	International			0	26	3	9								Y
Environmental Public Health Tracking Network Indicators [34]	H&W	≥ County	USA	Y		6	132	119	15		Y	Y	Y	Y		Y	Y
Environmental Quality Index [97]	Multiple	Neighborhood	Argentina		Y	1	15	0	7		Y		Y	Y			Y
Environmental Quality Index, EPA [98]	H&W	County	USA		Y	10	203	1	5		Y			Y			Y
Environmental Supports for Physical Activity Questionnaire [99]	Walkability/PA	Neighborhood	USA			0	5	0	12								Y
EPOCH Photo Neighborhood Evaluation Tool (EP-NET) [100]	Walkability/PA	Neighborhood	International			0	51	0	9								Y
European Livable Cities Index [39]	Liveability	City	Europe			3	11	3	6	Y	Y			Y			
EURO-PREVOB Community Questionnaire [101]	Multiple	Neighborhood	International			0	28	1	2				Y	Y			Y
EURO-URHIS Urban Health Indicators [102]	H&W	City	Europe			2	6	24	13	Y	Y			Y		Y	Y
FireStar Neighborhood Stability Framework [103]	Multiple	Neighborhood	Maryvale Village, Phoenix, AZ, USA			2	4	4	8					Y			
Flemish City Monitor [104]	Liveability	City	Flanders, Belgium			49	97	9	131		Y			Y		Y	
Glasgow Indicators Project [105]	H&W	City	Glasgow, Scotland			15	16	9	41				Y	Y	Y	Y	
Global City Indicators Facility - Your Health in the City Indicators [106]	H&W	City	International			Y	Y	Y	Y	Y	Y			Y		Y	
Global Liveable Cities Index [107]	Liveability	City	International			26	22	7	30	Y				Y			
Global Liveability Ranking [108]	Liveability	City	International			0	8	6	16	Y				Y			
Happy City Index [109]	Wellbeing	City	England			6	18	15	22		Y			Y		Y	
Health and Environmental Sustainability Indicators [110]	H&W	Neighborhood	Vietnam			0	11	4	1				Y	Y	Y	Y	
Health Determinants Indicators [111]	H&W	City	Japan			20	23	20	9								Y
Health Indicators Dashboard [112]	H&W	≥ City	Racine, WI, USA			3	14	31	9				Y				
Healthy Chicago 2.0 [113]	H&W	City and neighborhood	Chicago, IL, USA	Y	Y	3	4	38	30				Y			Y	
Healthy City Noise-Air Index [114]	H&W	City	International			0	5	0	0					Y			Y
Healthy Communities Index [115]	H&W	City	USA			2	4	0	4					Y			Y
Healthy Community Council Assessment [116]	Multiple	≥ City	Harrisonburg and Rockingham, VA, USA			3	9	11	17					Y		Y	
Healthy Resources Index [59]	H&W	City and neighborhood	Toronto, Canada		Y	0	3	0	1				Y	Y			
Housing and Environmental Quality Indicators [117]	Multiple	Neighborhood	Benin, Nigeria			1	21	1	2				Y	Y			Y
Indicators of Urban Ecosystem Health [118]	H&W	City and neighborhood	Canada			3	28	3	17								
Intra-city Social Well-Being Indicators [119]	Wellbeing	Neighborhood	Tampa, FL, USA		Y	11	15	8	13				Y	Y			
Irvine-Minnesota Inventory [120]	Walkability/PA	<Neighborhood	USA			0	60	0	10								Y
ISO 37120 [121]	Quality of life	City	International			12	56	9	23					Y		Y	
Kansas Health Matters [122]	H&W	County	Kansas, USA	Y	Y	15	24	76	23			Y	Y	Y	Y	Y	Y
Livability Index [47]	Liveability	Multiple	USA	Y		6	36	6	18		Y	Y	Y	Y	Y		
Livable Index System [123]	Liveability	Neighborhood	Tiexi District, Shenyang, China			0	18	1	13					Y		Y	
Liveability Assessment Tool [124]	Liveability	Neighborhood	Hunter New England, Australia			4	62	20	81				Y	Y		Y	Y
Local Climate Change Environmental Public Health Indicators (EPHI) [125]	H&W	≥ Neighborhood	International	Y		U	U	U	U				Y	Y		Y	
Local Health [126]	H&W	≥ Neighborhood	England	Y		8	2	50	25			Y	Y	Y	Y		
London Quality of Life Indicators [44]	Quality of life	City	London, UK			8	13	1	11					Y		Y	
London Ward Well-Being Scores [127]	Wellbeing	Neighborhood	London, UK	Y		2	3	2	5			Y	Y	Y			
London’s Health Strategy High Level Indicators [128]	H&W	City	London, UK			2	2	4	2						Y	Y	
Maryland Inventory of Urban Design Quality (MIUDQ) [129]	Walkability/PA	<Neighborhood	USA			0	26	0	1								Y
Multiple Environmental Deprivation Index (MEDIx) [130]	H&W	Neighborhood	UK		Y	0	8	0	0		Y		Y	Y			Y
Neighborhood Environment Walkability Scale (NEWS) [131]	Walkability/PA	Neighborhood	International			0	38	0	7								Y
Neighborhood Health Profile Reports [132]	H&W	City and neighborhood	Baltimore, MD, USA		Y	2	11	6	11				Y	Y	Y		
Neighborhood Design Characteristics Checklist (NeDeCC) [133]	H&W	Neighborhood	England			0	22	0	2								Y
Neighborhood Environment Indices [134]	Walkability/PA	Neighborhood	Putrajaya, Malaysia			0	4	0	0				Y				Y
Neighborhood Quality Index [135]	H&W	Neighborhood	Taiwan			0	1	0	15				Y	Y			Y
New Zealand Quality of Life Project [136]	Quality of life	City	New Zealand			41	58	27	87		Y			Y		Y	
New Zealand Systematic Pedestrian and Cycling Environmental Scan (NZ SPACES) [137]	Walkability/PA	<Neighborhood	New Zealand			0	47	0	7								Y
Objective and Subjective Quality of Life Indicators for Taiwan [138]	Quality of life	≥ City	Taiwan			9	5	5	7		Y						
Ottawa Neighborhood Study Indicators [139]	H&W	Neighborhood	Ottawa, Canada	Y	Y	Y	Y	Y	Y				Y	Y	Y		Y
Pasadena Quality of Life Index [140]	Quality of life	City	Pasadena/Altadena, CA, USA		Y	7	20	26	18		Y	Y		Y	Y	Y	Y
Pedestrian Environment Data Scan (PEDS) [141]	Walkability/PA	<Neighborhood	USA		Y	0	36	0	1					Y			Y
Peg Well-being Indicators [42]	Wellbeing	City and neighborhood	Winnipeg, Canada	Y		20	23	17	28				Y	Y	Y	Y	
Physical Activity Neighborhood Environment Scale (PANES) [142]	Walkability/PA	Neighborhood	International			0	14	0	3					Y			Y
Pilot Environmental Public Health Indicators [143]	H&W	County	USA			0	2	2	0							Y	Y
Places Rated Almanac [144]	Quality of life	City	USA		Y	1	15	7	21		Y				Y		
Plan for a Healthy LA Health Atlas/Health Profiles [43]	H&W	City and neighborhood	Los Angeles, CA, USA	Y	Y	21	44	21	26				Y	Y	Y	Y	
Proposed indicators linking health and sustainability [26]	H&W	City	International			0	15	5	4					Y	Y	Y	
Proxy Environmental Health Indicators for Accra [14]	H&W	Neighborhood	Accra, Ghana			1	69	22	13				Y	Y	Y		
Quality of Life Counts (Local) [10]	Quality of life	City	UK			5	17	1	7		Y	Y		Y	Y	Y	Y
Quality of Life in South East Queensland [145]	Quality of life	≥ City	South East Queensland, Australia			0	10	2	8								Y
Quality of Life in the City of Florence [146]	Quality of life	City and neighborhood	Florence, Italy			2	9	0	8				Y	Y		Y	
Quality of Life Index for Urban Transitional Neighborhood [147]	Quality of life	Neighborhood	Darvazeshemiran, Tehran, Iran			7	22	4	21					Y			Y
Quality of Life Index in Delhi [148]	Quality of life	City and neighborhood	Delhi, India		Y	2	24	1	5				Y	Y			
Quality of Life Indicator Program for San Diego-Tijuana Metropolitan Region [149]	Quality of life	City	San Diego-Tijuana, USA and Mexico			2	17	9	8	Y			Y	Y	Y	Y	Y
Quality of Life Indicators for Galway [37]	Quality of life	City and neighborhood	Galway, Ireland			4	9	0	7					Y	Y	Y	
Quality of Life Indicators for Thessaloniki [46]	Quality of life	City	Thessaloniki, Greece			10	21	6	19				Y	Y	Y	Y	
Quality of Life Reporting System [150]	Quality of life	≥ City	Canada			20	24	10	33		Y	Y		Y		Y	
Quality of Life Survey in Istanbul [151]	Quality of life	Neighborhood	Istanbul, Turkey			2	9	1	6								Y
Quality of Living Index [152]	Quality of life	City	International			2	13	2	13	Y	Y			Y			
Quality of Pedestrian Level of Service [153]	Walkability/PA	City	International		Y	0	4	0	1					Y			
Quality of Urban Life Assessment Tool [154]	Quality of life	Neighborhood	Doha, Qatar			0	83	0	28					Y			Y
Quality of Urban Life Index [36]	Quality of life	City	Atlanta, GA, USA		Y	5	7	3	5		Y			Y			
Residential Environment Assessment Tool [155]	Multiple	<Neighborhood	Wales			2	9	0	17					Y			Y
Richmond Health and Wellness Element Indicators [156]	H&W	City and neighborhood	Richmond, CA, USA			6	21	26	22				Y	Y	Y	Y	
Richmond Health Equity Indicators [157]	H&W	City	Richmond, CA, USA			6	17	23	26		Y			Y	Y	Y	
San Francisco Indicator Project [158]	H&W	City and neighborhood	San Francisco, CA, USA	Y	Y	18	46	8	35				Y	Y	Y	Y	Y
Scientific Assessment Standards of Livable Cities [159]	Liveability	City	China			6	13	1	13		Y			Y			
Seattle Healthy Living Assessment [160]	H&W	Neighborhood	Seattle, WA, USA			0	15	0	2					Y	Y	Y	
South Lanarkshire Index of Multiple Environmental Deprivation (SLIMED) [161]	H&W	Neighborhood	South Lanarkshire, Scotland		Y	0	7	1	1				Y	Y			Y
SPOTLIGHT Virtual Audit Tool [162]	Walkability/PA	Neighborhood	Europe			0	34	0	6								Y
Subjective Community Well-Being Indicator [163]	Wellbeing	City	Emilia-Romagna, Italy		Y	4	4	1	14		Y			Y			Y
Systematic Pedestrian and Cycling Environmental Scan (SPACES) [164]	Walkability/PA	<Neighborhood	Australia			0	33	0	6								Y
Think Health LA Indicators [165]	H&W	Multiple	Los Angeles, CA, USA	Y	Y	14	30	150	40			Y	Y	Y	Y		
Truckee Meadows Tomorrow [166]	Quality of life	City	Truckee Meadows, NV, USA			15	27	19	60					Y	Y	Y	
Urban Health Equity Assessment and Response Tool (Urban HEART) [167]	H&W	City and neighborhood	International			4	8	23	7			Y	Y	Y		Y	
Urban Health Equity Indicators for Mathare Informal Settlement [16]	H&W	Neighborhood	Nairobi, Kenya			3	9	2	3					Y	Y		
Urban Health Indicators for London [168]	H&W	<Neighborhood	London, UK		Y	1	2	0	1				Y	Y			Y
Urban Quality of Life in Switzerland [169]	Quality of life	City and neighborhood	Switzerland	Y		0	7	0	8					Y	Y	Y	
Vulnerability Indices [170]	H&W	Neighborhood	Worcester, MA, USA		Y	1	12	1	3				Y	Y	Y		Y
Walk Score [171]	H&W	Multiple	International	Y	Y	0	3	0	0		Y		Y	Y	Y		Y
Walkability Index [172]	Walkability/PA	City and neighborhood	USA		Y	0	3	0	1				Y	Y		Y	Y
Walkability Index (Bradshaw) [41]	Walkability/PA	Neighborhood	USA			0	6	0	4					Y			
Wellbeing Index [173]	Wellbeing	City	Santa Monica, CA, USA			11	10	13	44					Y	Y	Y	
West County Indicators Project [174]	H&W	Neighborhood	Richmond, CA, USA		Y	2	7	0	2					Y	Y		Y
WHO Environmental Health Indicators [175]	H&W	≥ City	Europe			2	34	7	1	Y				Y		Y	
WHO Healthy City Indicators [176]	H&W	City	International			3	14	11	4	Y	Y		Y	Y		Y	
Wholeness Index [177]	Quality of life	City and neighborhood	Dallas, TX, USA		Y	4	3	1	4				Y	Y	Y	Y	
Wisconsin Assessment of the Built Environment (WASABE) [178]	Multiple	Neighborhood	USA			2	17	0	10				Y	Y		Y	Y
World Health Organization Quality of Life (WHOQOL-100) [179]	Quality of life	<Neighborhood	International			8	12	16	65					Y		Y	Y
World Health Organization Quality of Life (WHOQOL-BREF) [180]	Quality of life	<Neighborhood	International			1	3	4	18					Y		Y	Y

*Y* yes, *U* unknown, *H&W* health and wellbeing, *PA* physical activity
