# Supplementary material for: Urban Health Indicator Tools of the Physical Environment: a Systematic Review
Source: J Urban Health. 2018 Apr 16;95(5):613–46. doi: 10.1007/s11524-018-0228-8 (PMC6181826; doi:10.1007/s11524-018-0228-8)
Supplement: Supplementary file 1 — (DOCX 122 kb) [file 11524_2018_228_MOESM1_ESM.docx]

# Supplementary Material

This document reports additional data and graphs about urban health indicator (UHI) tool characteristics. Headings relate to UHI tool characteristics listed in the review protocol.

## Producer

Figure 1 reports the types of organisations which produced UHI tools, ranging from international research collaborations to individual community groups. Research institutions were the largest producer of UHI tools (54.5%, 79/145), although their role as producers is reduced in those tools used beyond research purposes (29.1%, 23/79). City government(s) and non-profit partnerships/organisations developed a larger portion of these tools (19.0%, 15/79; 13.9% 11/79; and 12.7%, 10/79 respectively).


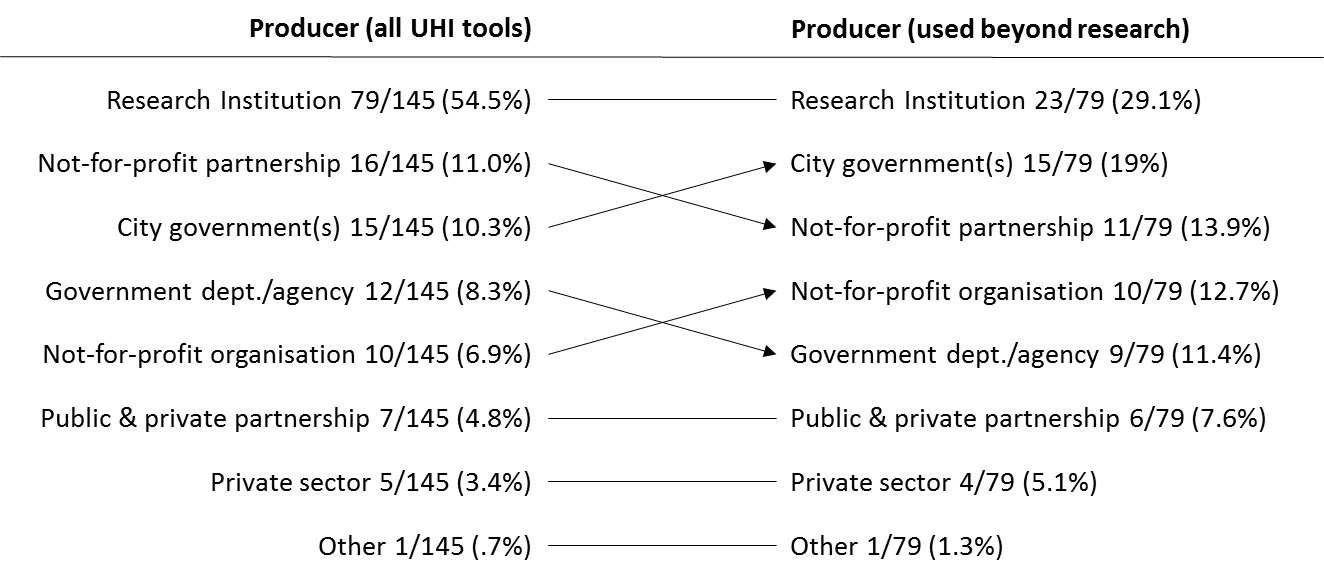


**Fig. 1** - Producers of UHI tools by organisation type, comparing all UHI tools with those used beyond research.

## Funder

Figure 2 shows the number of UHI tools funded by different organisation types. The private sector is rarely stated as a funder. Government agencies/bodies were involved in funding 26.2% of all UHI tools (38/145) and 35.4% of those used beyond research (28/79). The amount of funding was not usually stated.

Although research grants were often listed in academic papers, it was not always possible to find the associated funding amount. Three projects reported costs. The EURO-URHIS Urban Health Indicators project, part 2, had a total cost of EUR 3.6 million.[1] The annual cost of administering one resident Bristol’s Quality of Life survey was $20,000.[2] Developing the Multiple Environmental Deprivation Index (MEDIx) indicators was GBP 74,366.[3] On the basis of this limited data it is not possible to estimate an average cost for the development of new indicator tools.


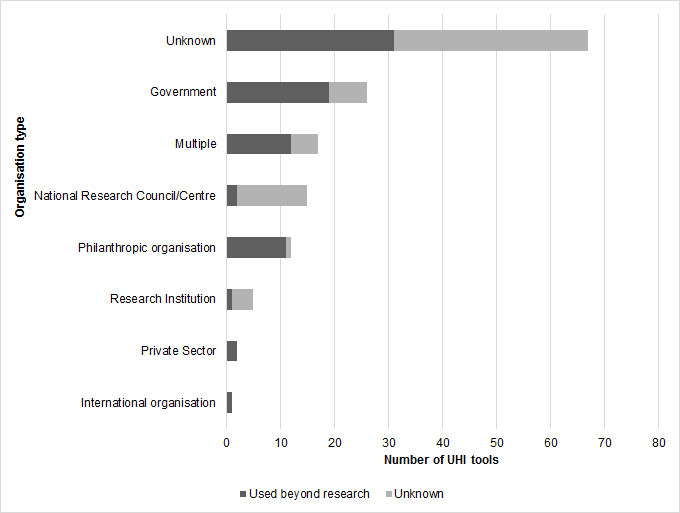


Figure 2 - Number of UHI tools funded by types of organisation, comparing UHI tools used beyond research and those for which their use beyond research is unknown.

## Geography

Geography refers to the scale at which particular UHI tools can be accessed. Figure 3 shows the number of tools which are available in various general geographic scales. A large number of tools (41%, 59/145) are available in individual cities, with national systems following closely behind.


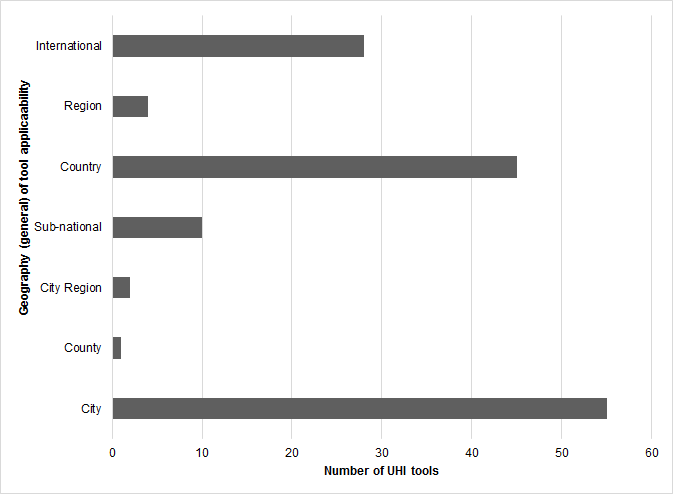


Figure 3 - Number of UHI tools at general geographic scales.

## UHI tool methodology

Some information about the methodology used to create the UHI tool was reported in 126/145 (86.9%) of tools. This included a range of potential points including: conceptual considerations, development process, community involvement, indicator selection, evidence-base, data sources, and weighting.

## Evidence-base

The majority of UHI tools (68.3%, 99/145) referred to an evidence-base which informed the methodology and/or indicator selection. Peer-reviewed literature was the largest primary source of evidence (used in 52.4%, 76/145 tools) followed by a small number of tools which used reviews of existing metrics/indicators (6), expert input (6), community input (5), and a combination of community and expert input (6).

## Weighting

The majority of tools (63.4%, 92/145) did not produce an index (or composite indicator). 30.3% (44/145) of UHI tools reported using a weighting system. Of these, 27.3% (12/44) were equally weighted. A number of approaches were taken to derive weighting systems, including statistical methods (such as Z-scores or Principal Component Analysis), expert input (such as Delphi Method or Analytical Hierarchy Process), community input, or user-determined weightings.

## Uncertainty

The issue of uncertainty was rarely discussed in the UHI tool methodologies, with only 16 tools mentioning the term. The context of uncertainty can be summarised as the uncertainty related to: the concept of community wellbeing (1 instance), measuring exposure (3 instances), methods of indicator selection (3 instances), risk assessment of exposure (1 instance), small population sizes (3 instances), and not relevant (4 instances).

## Main source of data

The majority of tools (57.9%, 84/145) used existing datasets from multiple organisations. Figure 4 shows that resident surveys (questionnaires or door-to-door) were much more likely to be used to gather data for subjective indicators (or tools which contained both subjective and objective indicators). Field audits (measurement of the urban environment by trained auditors) were more than twice as likely to be used for walkability/physical activity UHI tools than health and wellbeing tools or tools which measured multiple topics. Remote sensing, sensors and GIS or other maps were used by very few tools (3, 1 and 2 respectively).


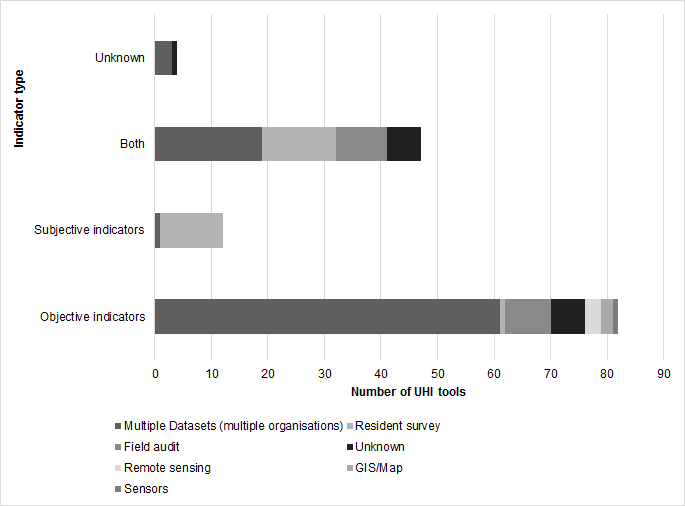


Figure 4 - Primary source of data by indicator type.

*References*

1. Euro-URHIS: Final Report. The Euro-URHIS Project. 2008.

2. Shepherd S, McMahon S. The Importance of Local Information: Quality of Life Indicators in Bristol. In: Sirgy PMJ, Phillips DR, Rahtz PDR, editors. Community Quality-of-Life Indicators: Best Cases IV. Springer Netherlands; 2009:111–20.

3. Richardson EA, Mitchell R, Shortt NK, Pearce J, Dawson TP. Developing Summary Measures of Health-Related Multiple Physical Environmental Deprivation for Epidemiological Research. Environ Plan A. 2010;42:1650–68.
